# Constructing tridimensional modeling and radiographic evaluation of diaphyseal fractures in a canine femur for veterinary education

**DOI:** 10.1590/acb400425

**Published:** 2025-01-13

**Authors:** Kleber dos Anjos Lucas, Rodrigo Gomes de Souza, Siham Kassab, Marco Aurélio Pereira Sampaio, Nongnuch Inpanbutr, Yuri Karaccas de Carvalho

**Affiliations:** 1 Universidade Federal do Acre – Biological and Natural Sciences Center – Rio Branco (AC) – Brazil.; 2 Universidade do Estado do Rio de Janeiro – Urogenital Research Unit – Rio de Janeiro (RJ) – Brazil.; 3 Universidade Federal Fluminense – Department of Morphology – Niterói (RJ) – Brazil.; 4 Ohio State University – Department of Veterinary Clinical Sciences – Columbus (OH) – United States of America.; 5 Universidade Federal Fluminense – Department of Pathology and Veterinary Clinic – Niterói (RJ) – Brazil.

**Keywords:** Printing, Three-Dimensional, Anatomy, Orthopedics, Teaching Materials, Femur, Femoral Fractures

## Abstract

**Purpose:**

To create tridimensional (3D) anatomical models of diaphyseal fractures in dogs (3D AMDFD) and to evaluate the models from their radiographs.

**Methods:**

The study consisted of six stages: preparation of femur from a healthy dog cadaver; digitalization of the bone through a 3D scanner and creation of the base model; creation of a 3D AMDFD based on the image of the base model, 3D modeling carried out to reproduce five different types of diaphyseal fractures; printing the models produced on a 3D printer with a thermoplastic material; insertion of neodymium magnets in the fracture line to allow the assembly and disassembly of the parts; and radiography of 3D AMDFD in lateromedial and craniocaudal positions.

**Results:**

The base model and 3D AMDFD had high precision in the replication of bone structures, like the bone in natura. The radiopacity and radiolucency of the 3D AMDFD did not necessarily correspond to the bone densities found in the radiography of the natural canine femur.

**Conclusion:**

The 3D AMDFD and their respective radiographs accurately reproduced the anatomical structures and fracture lines.

## Introduction

Bone fractures have a prominent role in the clinical-surgical routine in veterinary medicine^1^^–^ 3. Generally, most injuries occur due to car accidents, which account for about 80% of cases of fracture in dogs^3^^,^ 4. Long bone fractures in dogs correspond to 45% of the cases presented, of which 20 to 25% specially affect the femur^5^. Diaphysis fractures are defined as interrupting the continuity of the cortical bone and are usually caused by trauma. Femoral fractures in dogs in the diaphyseal portion correspond to 28% of cases^6^. Based on the direction line, diaphyseal fractures are classified as transverse, oblique, spiral, reducible comminuted, and irreducible comminuted. The diagnosis of diaphyseal fractures is made by the trauma history, physical examination, and mainly by radiographs in at least two positions^7^^,^ 8. The knowledge of these types of fractures is an essential component in the training of a veterinarian, as it requires that they know how to precisely recognize the fracture in order to provide the best clinical and surgical treatment^7^.

In this sense, tridimensional (3D) technology has been used in veterinary medicine as an innovative tool to aid in making the models for surgical planning^9^, in the medical clinic^10^ and orthopedic treatments^11^.

Tridimensional technology can favor the direct relationship with anatomical detail and put forward an alternative to the use of animals. Furthermore, this tool has great potential to provide a source of high-quality teaching materials^12^^–^ 15.

This research aimed to create 3D anatomical models of diaphyseal fractures in dogs (3D AMDFD) and to evaluate validity of the models from their radiographs.

## Methods

Cadaver femur from healthy, 5-year-old, mixed-breed dogs without concomitant injuries died from natural causes were macerated and prepared for further scanning manipulation and 3D printing.

The study was registered and approved under process number 23107.007273/2017-49 by the Ethics Committee on the Use of Animals of the Universidade Federal do Acre.

**Figure 1:**
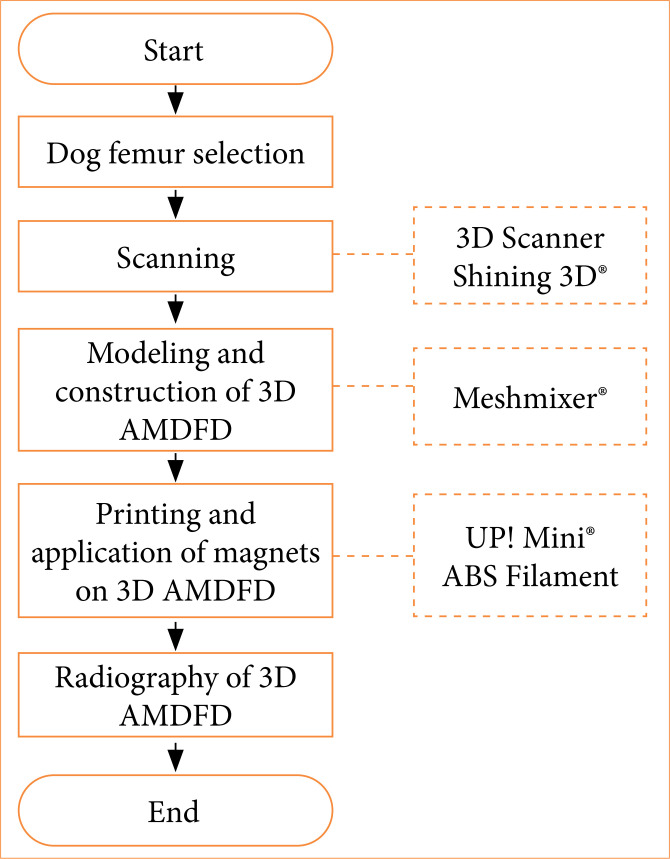
Flowchart of tridimensional (3D) canine diaphyseal fractures model creation.

The digital file obtained from scanning the *in-natura* bone served to build the 3D-base model. Thereafter, this was used to generate the 3D-AMDFD and represent the classification of diaphyseal fracture (Fig. 1).

3D AMDFD: tridimensional anatomical models of diaphyseal fractures in dogs. Source: Elaborated by the authors.

Scanning of the femur was performed using a 3D Scanner, Model EinScan-SP (Shining 3D^®^, Zhejiang, China), using the EinScan-SP Version 2.6.0.8 software included in the equipment.

Images were saved in *.stl* format and stored in a database. Subsequently, they were transferred to a 3D creation and manipulation system, Autodesk Meshmixer, version 3.1 (Autodesk Inc., California, United States of America), for modeling and composition of the 3D AMDFD. The modeling stage consisted of separating the anatomical regions in which fractures occur. There was no loss of information during this process, and all structures of the femur were maintained.

Fracture locations reproduced in the 3D AMDFD were based on a study by DeCamp et al.^16^ e Fossum^17^ (Table 1).

**Table 1:** Classification of diaphyseal fractures by direction.

Classification	Features
Transverse	Fracture line forms a right angle with the long axis of the bone.
Oblique	Fracture line forms an oblique angle with the long axis of the bone.
Spiral	Fracture line forms spiral curve around the bone.
Reducible comminutive	Fracture line does not communicate each other and has three or more fragments.
Irreducible comminutive	Fracture line communicates with each other and has many fragments.

Source: adapted from DeCamp et al.^16^ e Fossum^17^.

The constituent parts of each 3D AMDFD were printed using UP 3D Mini (Beijing Tiertime Technology Co. Ltd., Beijing, China), which uses fine quality fused deposition modeling technology and acrylonitrile butadiene styrene (ABS)-grade thermoplastic material, with a 99% internal fill and a layer thickness of 0.15 mm. After printing, manual finishing was performed. Neodymium magnets, 4 mm in diameter and 2 mm in height, were inserted in the fracture lines of each segment of the model, to enable assembly and disassembly of the parts.

### Radiographic study of 3D AMDFD

After completion of the construction phase, models were taken to the diagnostic imaging center for radiographing. 3D AMDFD were radiographed using Emic Limex with a radiation intensity of 48 kV and exposure time of 3 seconds.

The models were focused in two positions: craniocaudal (CC) and lateromedial (LM), as recommended by Fossum^17^ for fractures in long bone. Images were edited using Carestream Image Suite 4.0.

For the radiographic examination, it was necessary to print the models once again with 99% internal filling and without magnetic neodymium. In this preparation, a transparent double-sided adhesive tape was inserted in the fracture surfaces of the models.

## Results

**Figure 2:**
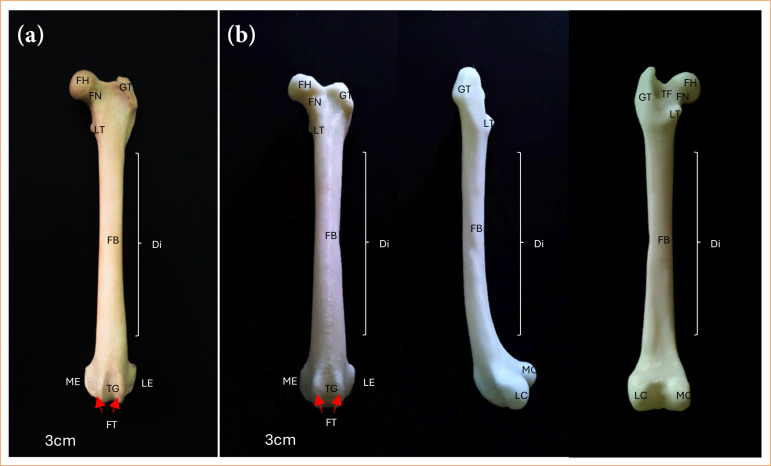
Femur of the dog, **(a)** *in-natura* – cranial view; **(b)** 3D base model – cranial, lateral and caudal views, respectively.

The 3D base model of the femur showed a similar conformation as the natural bone, maintaining the same length and width, in addition to reproducing the structures that identify the bone. The following anatomical structures were observed: femoral head; femoral neck; greater trochanter; lesser trochanter; trochanteric fossa; femoral body; trochlear groove; femoral trochlea; lateral epicondyle; medial epicondyle; lateral condyle; and medial condyle (Fig. 2).

3D: tridimensional; FH: femoral head; FN: femoral neck; GT: greater trochanter; LT: lesser trochanter; FB: femoral body; TG: trochlear groove; FT: femoral trochlea; ME: medial epicondyle; LE: lateral epicondyle; Di: diaphysis; TF: trochanteric fossa; LC: lateral condyle; MC: medial condyle. Source: Elaborated by the authors.

It was necessary only 5 minutes to step of scanning. The amount of time spent for creation, printing time, amount of material used in manufacturing, and final printing costs of 3D AMDFD were recorded (Table 2).

**Table 2:** Creation time, print time, quantity of material used, and costs of the 3D AMDFD.

3D AMDFD	Creation time (h)	Print time (h)	Material used (g)	Cost (US$)
Transverse	0.5	3.6	17.3	0.52
Oblique	0.5	4.4	20.7	0.62
Spiral	0.7	4.1	19.1	0.58
Reducible Comminuted	1.0	3.9	17.8	0.53
Irreducible Comminuted	1.5	4.1	18.4	0.55
TOTAL (3D AMDFD)	4.2	20.1	93.3	2.80

3D AMDFD: tridimensional anatomical models of diaphyseal fractures in dogs. Source: Elaborated by the authors.

The modeling of individual fracture types resulted in different times during the creation process. The models of spiral fracture, reducible comminuted, and irreducible comminuted obtained more time, due to the complexity of creating fracture lines. Furthermore, transversal and oblique fractures resulted in a shorter time, since the fracture lines in these two types are represented by a single cut.

Printing costs were represented by the amount of filament used, which corresponded to 70% of the total value. The other 30% of the cost was attributed to the depreciation of the machine and the consumption of electricity. However, equipment costs (3D printer and 3D scanner) were not accounted for.

**Figure 3:**
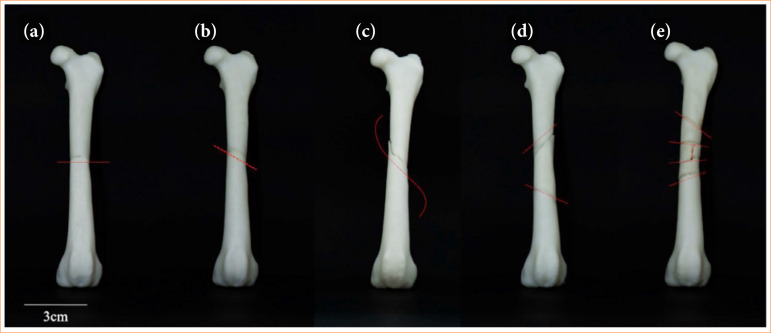
Tridimensional of different anatomical models of diaphyseal fractures in dogs. **(a)** Transverse; **(b)** oblique; **(c)** spiral; **(d)** reducible comminuted; **(e)** irreducible comminuted. Red lines indicate fracture foci.

Source: Elaborated by the authors.

Five 3D AMDFD were printed, each representing the different diaphyseal fractures in the canine femur (Fig. 3).

The *3D AMDFD-Transverse* was cut transversely in relation to the longitudinal axis of the bone, resulting in two portions. A fitting was made in each portion to place the magnet in order to be able to be assembled and disassembled.

The *3D AMDFD-Oblique* fracture was made by a diagonal cut in the body of the femur resulting in two portions. Two grooves were created in each portion for better stability and firmness of articulation (Fig. 3b).

In the *3D AMDFD-Spiral*, a spiral cut was made in the body of the femur, resembling a twist, which resulted in two portions. Three inserts were created in each portion of the bone for the introduction of the magnet and for better stability (Fig. 3c).

For the *3D AMDFD-Comminuted Reducible*, two cuts were made, separated and in irregular directions, resulting in three segments, which characterize this type of fracture. Two slots were created in each portion for the insertion of the magnet in order to have good stability.

The *3D AMDFD-Comminuted Irreducible* was made several cuts, some separate and others that are found, mimicking full and partial fracture lines, resulting in six portions, irregular, segmental of different sizes, in order to characterize this type of fracture. A slot was created in each portion of the fracture in different sizes, each slot compatible with a magnet and with the size of the surface of the fracture portion.

**Figure 4:**
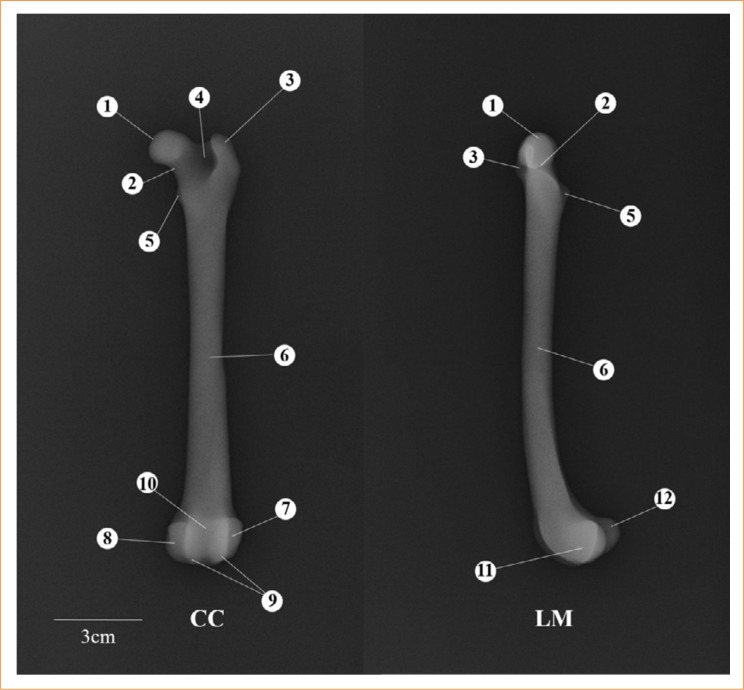
Radiographs of tridimensional base model of the dog femur. Craniocaudal (CC) and Lateromedial (LM) views. 1) Femoral head; 2) femoral neck; 3) greater trochanter; 4) trochanteric fossa; 5) lesser trochanter; 6) femoral body; 7) lateral epicondyle; 8) medial epicondyle; 9) femoral trochlea; 10) trochlear groove; 11) lateral condyle; 12) medial condyle.

Source: Elaborated by the authors.

The radiographic images of the 3D AMDFD showed clear radiopaque and radiolucent regions that made it possible to observe the different anatomical structures corresponding to the femur: femoral head; femoral neck; greater trochanter; lesser trochanter; trochanteric fossa; femoral body; lateral epicondyle; medial epicondyle; femoral trochlea; trochlear groove; lateral condyle; and medial condyle (Fig. 4). In all 3D AMDFD radiographs, it was not possible to differentiate the cortical and medullary regions since the internal padding used for printing was 99%.

The radiographs of the *3D AMDFD-Transverse* showed a fracture line evident in the CC and LM positions. The radiographs of the *3D AMDFD-Oblique* showed the fracture line evident in the CC position and in the LM position. It was possible to identify the beginning and the end of the fracture line.

The X-ray of the *3D AMDFD-Spiral* showed a fracture line evident in the CC position. It was difficult to identify the type of fracture, but possible to observe only the beginning and the end of the fracture line in the LM view.

Radiographs of the *3D AMDFD-Comminuted Reducible* CC position made it possible to perfectly evidence the fracture lines, whereas in the LM position it was possible to visualize only the beginning and end of the proximal and distal fracture lines.

The radiographs of the *3D AMDFD-Comminuted Irreducible* showed evident fracture lines that were easily observed both in the CC and LM positions.

**Figure 5:**
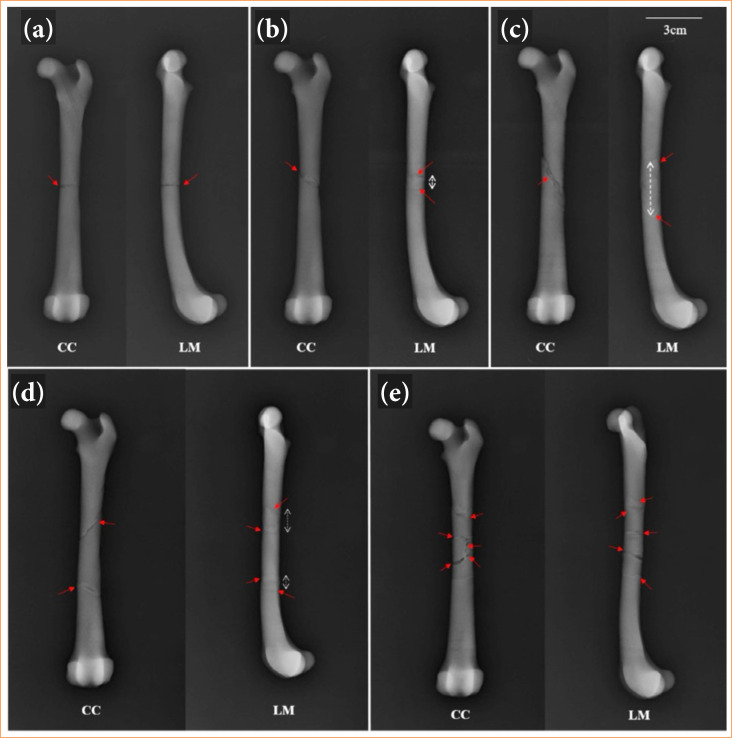
Radiographs of 3D AMDFD of the dog femur. Craniocaudal (CC) and Lateromedial (LM) views. **(a)** Transverse; **(b)** oblique; **(c)** spiral; **(d)** reducible comminuted; **(e)** irreducible comminuted. Red arrows indicate fracture foci, white arrows indicate the beginning and end of the fracture.

The neodymium magnets were removed from the models so as not to produce artifacts, which can hinder or prevent the observation of fracture lines on the models’ radiographs. In place, a transparent double-sided tape was introduced in the fracture lines of the models to fix the fragments (Fig. 5).

3D AMDFD: tridimensional anatomical models of diaphyseal fractures in dogs. Source: Elaborated by the authors.

In general, in radiographs of the *3D AMDFD-Transversal and Comminuted Irreducible model*, it was evident the visualization of the fracture lines in the CC and LM positions.

In the *3D AMDFD-Oblique, Spiral and Reducible Comminuted* model, it was possible to observe the fracture lines in the CC position, but with difficulty in the LM position. In the LM position, it was possible to observe only the begin and end of the proximal and distal fracture lines.

## Discussion

Creation of 3D AMDFD in this study was based on the need to represent diaphyseal fractures in dogs’ femur, which is relevant in veterinary medicine^3^^,^ 11. This may be due in part to limitation of educational materials or failures of teaching institutions in prioritizing and enforcing education in this field^18^.

The digitalization of dogs’ femur *in natura* was a fundamental step in the research in order to obtain a digital file which preserved anatomical as referred in the base model. Similarly, other studies describe the digitalization by 3D scanners as a crucial step in the development of anatomic models^19^^–^ 21.

Although we achieved good anatomical representation of the dog’s femur in our base model, we also observed structures that were not sufficiently reproduced to serve as anatomical references. Among them, there was the poor representation of lateral and medial lips, lateral and medial supracondylar tuberosities, and intercondyloid fossa. These findings are similar to those reported by Li et al.^21^, in which it was possible to visualize the nutrient foramina of bovine bones (femur and cervical vertebra) in a digital file, but these structures were not reliably reproduced when printed. Another limitation of the base model was non-visualization of the medullary cavity. This limitation is directly related to the method of obtaining the images, since the 3D scanner captures only the surface of the femur^22^^–^ 24.

The poor visualization of the lateral and medial lips, lateral and medial supracondylar tuberosities, and intercondyloid fossa did not affect viability of the 3D AMDFD. These findings were comparable to the study of Thomas et al.^20^, which reproduced dogfish and cane toad skeletons, but lost some foramina and details of bone mass. Moreover, the absence of these details did not detract from the proposal to teach anatomy through 3D impressions.

The total time’s creation of 3D AMDFD was 4.2 hours. This time was relatively short in comparison with the study of Tacher et al.^25^, which created a dog’s brain from computed tomography, with the total time of 12 hours. The difference in the creation’s time can be justified to fractures’ foci of 3D AMDFD were represent only to cuts in the diaphyseal portion and digitalization fast promoted by 3D scanner utilized.

The creation’s time of the anatomical models of spiral, reducible comminuted, and irreducible comminuted fractures were greater when compared with the transverse and oblique fractures. This fact is explained by the degree of complexity in reproducing the fracture’s lines and the numbers of resulting parts for each model.

Even though the 3D AMDFD do not have many anatomical structures, the printing time for each model was high when compared with other more complex anatomical models, such as a dog’s skull with printing time of 7 hours^26^. These values can be justified due to settings used during printing. These included internal filling of the model, thickness Z, temperature, and extruder nozzle, which directly influence the time and costs of printing^27^.

To compare the total cost of our models with other studies, we considered the use of the same printing technique with similar materials. In the study by Li et al.^21^, 140.4 g of thermoplastic filament (PLA) was used for printing bovine femurs, and the reported cost was US$ 3.50 per unit. The material used in this study was also a thermoplastic filament (ABS), and 93.3 g of filaments were used, with the total cost of US$ 2.80 per unit. By comparison, even though printing different parts, we observed similarities in the type and amount of material used, and the cost of printing.

For the 3D AMDFD models to be feasible for teaching, manipulation, and demonstration of fractures, we chose to use neodymium magnets to detach parts of the models. This allows for visualization of the fracture foci surfaces. This method of articulation is like that one used by Lima et al.^15^, who reported that the presence of magnets in 3D models allowed separation of the pieces, and visualization of the fracture foci surfaces in 3D fractures models of dog mandibles.

The stability of 3D AMDFD fracture lines were assessed by the number and the size of each magnet used during manufactured. The 3D AMDFD Transverse parts were united with the presence of a single large magnet, but with the possibility of rotation between the parts of the model. This observed rotation is similar to the study by Dallabrida et al.^28^ when analyzing the biomechanics of osteosynthesis of transverse shaft fractures of femurs in dogs.

In the 3D AMDFD (oblique, spiral, comminuted reducible, and comminuted irreducible), we chose to place a magnet in the longitudinal cuts and two smaller magnets in the oblique and transversal cuts for stability between parts of the model. Additionally, we did not find prior study on positioning of magnets in articulated models. In this case, we supported positioning of the magnets using reports for surgical treatments of diaphyseal fractures^8^^,^ 29.

The use of 3D printed models has potential to provide a source of high-quality teaching material^30^. However, for these educational models to be scientifically valid, methods that objectively prove their quality are needed, and should not be based only on the perception of the users^12^^,^ 31. Keeping that fact in mind, we chose to perform 3D AMDFD radiographs.

For 3D AMDFD imaging, the CC and LM radiographic placements per model were enough. These findings agree with the study by Rubin et al.^29^, which analyzing radiographic images of canine to determine the incidence of pathological fractures associated with appendicular primary bone tumors in dogs in clinics.

The radiopacity and radiolucency of the 3D AMDFD did not necessarily correspond to the bone densities found in the radiography of the natural canine femur. This fact can be explained by the techniques used to obtain the images (3D scanner) and manufacture the models, the material used for printing (thermoplastic), and the printing parameters used. Another factor that may have influenced the image results is the radiographic technique used. Since the thermoplastic material is unconventional, the radiation intensity and exposure time were similar to that of Lima et al.^15^.

The 3D AMDFD were printed with maximum internal fill (99%). If you use an internal fill less than 99%, it will generate internal grids, which are visible on the radiographs in the models. The presence of internal grids are artifacts that block the visualization of the fracture lines of the models^15^.

The 3D AMDFD radiographic images represented the classification of the diaphyseal fractures in canine femur. However, the models were not representative in the case used with the finality of surgery training. It is not possible to reproduce different bone density of the compact and cancellous bone. Surgical training requires knowledge of bone density and forces dissipated along trabeculae of spongy bone to stabilize fracture with plates and screws^8^^,^ 32.

It was necessary to remove the neodymium magnets in the models for radiographic imaging, since in an initial test. These caused visual artifacts which interfered with visualization of the fracture foci. Similar findings were reported by Lima et al.^15^, when trying to X-ray 3D models of fracture of mandible in dogs with the presence of magnets.

The 3D canine femur had already been printed as a tool for teaching veterinary anatomy^18^. However, we proposed the use of 3D AMDFD for making radiographic images, to provide a valuable resource for the demonstration of radiographic aspects that are not usually covered in undergraduate studies.

It is noteworthy that the 3D creation methodology used differs from other research centers, since the 3D AMDFD were built from the scanning of the bone *in natura*. Subsequently, the radiographs of the models were generated. Commonly, studies point to the creation of 3D models from medical images^33^^,^ 34.

Digital files generated from 3D AMDFD can contribute to 3D educational model databases, so that any person or educational institution is able to access these models^24^. An example of this possibility is the model of the dog ear canal, which is available on the Thingiverse digital platform^35^.

## Conclusion

The 3D AMDFD and their respective radiographs reproduced the anatomical structures and fracture lines which characterize the disease. The models provide options to use for teaching in the disciplines such as anatomy, surgical anatomy, surgery, and radiology, which can also assist in clinical diagnosis. The impact these models can generate will manifest when using in veterinary medical education.

## Data Availability

All data sets were generated or analyzed in the current study.
